# A normative spatiotemporal MRI atlas of the fetal brain for automatic segmentation and analysis of early brain growth

**DOI:** 10.1038/s41598-017-00525-w

**Published:** 2017-03-28

**Authors:** Ali Gholipour, Caitlin K. Rollins, Clemente Velasco-Annis, Abdelhakim Ouaalam, Alireza Akhondi-Asl, Onur Afacan, Cynthia M. Ortinau, Sean Clancy, Catherine Limperopoulos, Edward Yang, Judy A. Estroff, Simon K. Warfield

**Affiliations:** 1 Boston Children’s Hospital and Harvard Medical School, Department of Radiology, Boston, MA 02115 USA; 2 Boston Children’s Hospital and Harvard Medical School, Department of Neurology, Boston, MA 02115 USA; 3 Boston Children’s Hospital and Harvard Medical School, Department of Anesthesia, Boston, MA 02115 USA; 4 Washington University School of Medicine in St. Louis, Department of Pediatrics, St. Louis, MO 63130 USA; 5 Children’s National Medical Center, Department of Diagnostic Imaging Radiology, Washington DC, 20010 USA

## Abstract

Longitudinal characterization of early brain growth *in-utero* has been limited by a number of challenges in fetal imaging, the rapid change in size, shape and volume of the developing brain, and the consequent lack of suitable algorithms for fetal brain image analysis. There is a need for an improved digital brain atlas of the spatiotemporal maturation of the fetal brain extending over the key developmental periods. We have developed an algorithm for construction of an unbiased four-dimensional atlas of the developing fetal brain by integrating symmetric diffeomorphic deformable registration in space with kernel regression in age. We applied this new algorithm to construct a spatiotemporal atlas from MRI of 81 normal fetuses scanned between 19 and 39 weeks of gestation and labeled the structures of the developing brain. We evaluated the use of this atlas and additional individual fetal brain MRI atlases for completely automatic multi-atlas segmentation of fetal brain MRI. The atlas is available online as a reference for anatomy and for registration and segmentation, to aid in connectivity analysis, and for groupwise and longitudinal analysis of early brain growth.

## Introduction

Early stages of human brain development are particularly important as any abnormality in development may result in long-term neurodevelopmental impairment and may even affect the survival in the perinatal period and later in childhood^1–10^. Precise characterization of neural development and cortical maturation encompassing neurogenic events such as cell proliferation, neuronal migration, and myelination may enable improved diagnosis, which is critical for appropriate prenatal counseling and medical treatment and intervention. In addition, adequate characterization of these developmental processes may provide insight into pathophysiology underlying neurological disorders such as autism and developmental delay, which are thought to begin in the very early stages of life^11^. In the future, these findings could even suggest therapeutic targets for interventions^12–15^. Postnatally, highly accurate *in-vivo* analysis is critical to the development of neural rescue therapies, such as brain hypothermia^3, 8, 16, 17^, and precise delineation of injury or malformation^4, 5, 7, 10^.

There have been numerous studies in the past decade on the analysis and characterization of early brain development by means of magnetic resonance imaging (MRI)^18–34^. The MRI study of the developing brain, however, has been much more difficult than the longitudinal studies of aging and disease progression in adults and children, because of (1) the challenges in scanning fetuses and neonates including fetal/maternal and neonatal motion and limited resolution, and (2) rapid changes in brain structure and function during the early stages of growth specifically during the third trimester of pregnancy. There has been a gap in technology and a critical need for imaging and image processing tools and resources, in specific digital brain atlases, that enable automatic segmentation, and volumetric, morphologic, longitudinal, and groupwise analysis of brain development based on *in-vivo* fetal MRI. Fetal MRI is challenged by intermittent fetal and maternal motion that disrupts the spatial encoding needed for 3D imaging, thus is limited to fast 2D slice acquisitions. A series of studies, however, have shown that fetal MRI may be reconstructed in 3D through retrospective inter-slice motion correction and volume reconstruction^35–42^. This evolving technology has enabled significant new advances in computational analysis of fetal brain MRI including atlas construction, automatic fetal brain MRI segmentation, and groupwise analysis^25–27, 43–48^.

Since the brain size, shape, and structure changes rapidly during the fetal and neonatal periods, atlases that cover these periods should be spatiotemporal (dynamic or 4D) rather than being static or 3D. The construction of digital spatiotemporal MRI atlases of early brain development is relatively new: Kuklisova-Murgasova *et al*.^49^ developed a 4D probabilistic atlas of early brain growth from *in-vivo* MRI of 142 preterm infants in the 29 to 44 weeks post-menstrual age. They used pairwise affine registration of anatomy with kernel regression in age for atlas construction. Serag *et al*.^46^ used a non-rigid registration approach based on Bspline free-form deformations (FFD)^50^ and showed a marked improvement over the use of affine registration in atlas construction. Makropoulos *et al*.^48^ used a similar approach to construct a probabilistic spatiotemporal atlas of the neonatal brain from 420 segmented MRIs of neonates (including preterm neonates) scanned between 27 to 45 weeks post-menstrual age. To improve the FFD-based 4D atlas construction framework, Schuh *et al*.^51^ developed diffeomorphic registration based on the Log-Euclidean mean of inverse consistent FFD transformations. MRI acquisition in the prematurely born baby is simpler than fetal MRI, as three dimensional imaging is possible due to the reduced motion of the preterm infant as compared to the fetus. However, key early brain development occurs before the age at which prematurely born infants are viable, and the factors which lead to premature birth may alter brain anatomy in a number of ways, including by direct injury such as stroke, and by delayed maturation. There is an increasing need for digital fetal brain MRI atlases that extend over the second and third trimesters of gestation in which the brain passes an exponential phase of growth.

The development of fetal brain MRI atlases is more difficult than neonatal atlases because of the challenges in high-quality fetal MRI acquisition and its 3D reconstruction and the rapid changes in brain anatomy and shape throughout the course of *in-utero* brain maturation. The first spatiotemporal probabilistic MRI atlas of the fetal brain^44^ was developed through polynomial fitting and non-rigid groupwise registration of manually segmented fetal brain tissue in 20 healthy fetuses in the GA range of 20.57 to 24.71 weeks. More recently the FFD-based atlas construction method^46^ was used to construct a spatiotemporal atlas of the fetal brain in the GA range of 22 to 38 weeks from MRI of 80 fetuses^52^. This atlas is available online through brain-development.org. There are a few other fetal brain atlases, such as a spatiotemporal latent atlas of the fetal brain in 20 to 30 weeks GA based on annotated fetal brain MRIs^53^, a spatiotemporal cortical surface atlas of the fetal brain through sulcal matching from MRI of 80 healthy fetuses^54^, and an *ex-vivo* spatiotemporal MRI atlas of the fetal brain in GA range of 15 to 22 weeks from 34 postmortem human fetal brains^55^. For a review of developmental brain atlases we refer to Gui *et al*.^56^.

The majority of previous works on fetal and neonatal atlases focused on the construction of probabilistic atlases which relied upon manual segmentations of original data^44, 48, 49, 52–54, 57^. In this study, we focused on the construction of a sharp deformable spatiotemporal atlas of the fetal brain to facilitate the use of a probabilistic label fusion approach for atlas labeling and segmentation. Affine or low-dimensional FFD transformations were used for probabilistic atlas construction in most of the earlier studies, however, the capacity of low-dimensional and non-diffeomorphic transformations is intrinsically limited in capturing the anatomical variability of the population for deformable atlas construction. This motivated the use of high-dimensional deformation models such as the FFD model^50^ and its diffeomorphic extensions^58, 59^ for spatiotemporal atlas construction^46, 51^. The inability to bring the same anatomy in to alignment across the group of subjects and across age is reflected in the lack of sharp boundaries. In this work we aimed to build a detail-preserving sharp anatomical atlas of the fetal brain that is an unbiased average representative of the anatomy at all key gestational ages. For this purpose, we integrated kernel regression in age with symmetric diffeomorphic deformable registration^60^ in space. Symmetric diffeomorphic deformations generate inverse consistent transformations that allow large deformations^61–63^. Every pair of source and target images are affected equally by the symmetric deformation and interpolation thus asymmetric bias is reduced. In contrast, in free-form deformations, deformations should be small otherwise invertability is not guaranteed. In our earlier work^64^ we examined different configurations and observed that a formulation based on symmetric diffeomorphic deformations outperformed alternative configurations based on FFD and Demons deformation models.

To provide an atlas as a useful resource for automatic segmentation and computational analysis of fetal brain MRI in this work we (1) constructed an *in-vivo* detail-preserving spatiotemporal MRI atlas of the fetal brain in the GA range of 21 to 37 weeks from *in-vivo* MRI of 81 healthy fetuses scanned in the GA range of 19 to 39 weeks, (2) labeled developing brain tissue and structures on the fetal brain MRI atlas, and (3) evaluated the use of this atlas and additional individual subject fetal brain MRI atlases in multi-atlas segmentation. The paper is organized as follow: In the materials and methods we describe our atlas construction method and the techniques we used to generate labels on our spatiotemporal and individual-subject atlases. The quality and sharpness of the constructed atlas helped us to effectively use a recently-developed probabilistic label fusion algorithm that combines intensity and local map images of multiple templates to train a local Gaussian mixture model^65^. We used this algorithm along with neonatal brain atlases^66, 67^ as a guide to initiate segmentations on late-gestation points of our spatiotemporal atlas and carried out extensive manual segmentations to generate fetal atlas labels. The results section presents the atlas and its labels and a segmentation evaluation. The atlases with labels are available online at one week intervals between 21 and 37 weeks of GA and can be generated at any given continuous age point within this range.

## Materials and Methods

### Imaging data

Fetal brain structural MRI is performed through repeated T2-weighted half-Fourier acquisition single shot fast spin echo (T2wSSFSE) scans in the orthogonal planes of the fetal brain^68^. The data for atlas construction in this study was obtained from fetal MRI of 81 healthy fetuses scanned at a GA between 19 and 39 weeks (mean = 30.1, stdev = 4.5). A different set of subjects were used for test. The test set was based on research MRI scans of 7 healthy fetuses in the GA range of 23 to 38 weeks (mean = 32.1, stdev = 5.4). Exclusion criteria were multiple-gestation pregnancy, maternal contraindication to MRI, known fetal congenital infection, fetus with brain or body abnormalities detected by prenatal ultrasound or MRI, and known chromosomal abnormalities by clinical genetic testing. All fetuses were scanned either by 3-Tesla Siemens Skyra or Trio MRI scanners (Siemens Healthineers, Erlangen, Germany) with 18-channel body matrix coils, or by a 1.5-Tesla Achieva scanner (Philips Medical System, Netherlands) with a 5-channel phased-array cardiac coil. Multi-planar repeated T2wSSFSE imaging was performed with a 2 or 4 interleaved acquisition, with effective echo time 100 and 120 ms, repetition time of 1400–2000 ms, variable field of view based on the fetal and maternal size, 2-mm slice thickness, no inter-slice gap, and 256 × 204, 256 × 256, or 320 × 320 acquisition matrices with in-plane resolutions between 0.9 and 1.1mm. The duration of MRI acquisitions for the images used in this study was 15 to 30 minutes. No maternal sedation was used. All methods and experiments were performed in accordance with relevant guidelines and regulations. The study was approved by the Boston Children’s Hospital Institutional Review Board and the Committee on Clinical Investigation and written informed consent was obtained from all participants.

### Pre-processing

**Figure 1:**
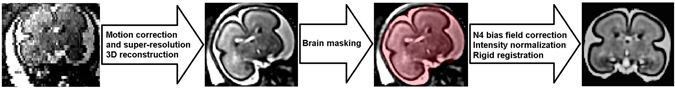
The preprocessing steps in fetal brain MRI analysis: (a) shows the out-of-plane view (coronal view) of an original axial T2wSSFSE scan, (b) is the volumetric image obtained from iterations of inter-slice motion correction and robust super-resolution volume reconstruction^38^, (c) shows the brain mask obtained through supervised levelset segmentation and manual refinement, and (d) is the reconstructed image, reoriented and co-registered to the common atlas coordinate space after N4 bias field correction and intensity normalization.

The preprocessing steps of volumetric fetal brain MRI reconstruction are shown in Fig. 1; where (a) shows a coronal view of an original axial T2wSSFSE scan, (b) shows the volumetric image obtained after 5 iterations of motion correction and robust super-resolution volume reconstruction^38^. Four to 15 (mean = 8) scans were used for reconstruction, for which the fetal brain region was cropped automatically using an ellipsoid mask manually placed on the brain region. The reconstruction processing time was between 1 and 20 hours depending on the size and number of the images and the amount of motion, and involved between 3 to 10 iterations of motion correction and super-resolution volume reconstruction using^38^ which was done automatically. (c) in this figure shows the brain mask obtained from supervised level set segmentation in itksnap^69^ followed by manual refinement. Manual refinement of brain masks took between 15 minutes to 2 hours for each case depending on the size and position of the fetal brain and the surrounding structures and the quality of the reconstruction, and (d) shows the image after intensity inhomogeneity correction and rigid alignment to the atlas space. Intensity inhomogeneity was corrected using the N4 algorithm^70^, which is an improved version of the nonparametric nonuniform intensity normalization (N3) algorithm^71^. The N4 algorithm was applied with brain masks and generated smooth bias fields that did not locally affect the appearance of small structures. The intensity range of the output images was normalized between subjects by linearly rescaling the intensities to match the maximum values corresponding to the CSF. In order to compensate for the non-orthogonal orientation of the fetal brain MRI after reconstruction, the reconstructed images were reoriented by using the direction cosines matrix of one of the original scans. The brain images were then registered to the anatomic space of the atlas through first order geometric moments matching followed by multi-scale mutual information based rigid registration as previously described^45^. The MRI scans of the 7 fetuses in the test set were reconstructed by volume reconstruction using^42^, and processed in a similar fashion, but were manually labeled using the manual labeling procedure that is described in the Atlas Labeling and Segmentation section.

### Atlas construction

We aimed to develop a four-dimensional (3D + time) atlas that characterizes normal fetal brain development *in-utero*. This spatiotemporal atlas should effectively capture and encode the anatomic variability of the population across gestation. To achieve this, we integrate kernel regression over age^72^ with symmetric diffeomorphic deformable registration based on a viscous fluid deformation model^60^ in space. A key characteristic of this approach, as compared to the spatiotemporal atlas construction methods recently applied to fetal brain MRI, is the use of diffeomorphic deformations that are smooth and invertible^62, 73^.

Given a collection of *M* images ${I}_{i}(x):{R}^{3}\to R$ acquired at the corresponding GA *t*_*i*_ from fetuses in the population, we formulate the problem as finding a set of transformations ${h}_{i}:{R}^{3}\to {R}^{3}$ and a template $I(x,t):{R}^{4}\to R$ that is a weighted minimum distance representation of the population anatomy at any given age *t*. The problem is formulated as:

1 $$({\hat{h}}_{i},\hat{I}(t))=argmi{n}_{{h}_{i},I}\frac{1}{{\Sigma }_{i\mathrm{=1}}^{M}{\bf{K}}(t-{t}_{i})}{\Sigma }_{i\mathrm{=1}}^{M}{\bf{K}}(t-{t}_{i})[E(I(t),{I}_{i},{h}_{i})+{\Vert L{v}_{i}\Vert }^{2}],$$

where **K** is the Gaussian kernel, *E* is a cost function defined based on the inverse of the similarity of two images, and

2 $$\frac{d}{ds}{h}_{i}(x,s)={v}_{i}({h}_{i}(x,s),s);\,s\in \mathrm{[0,\; 1].}$$

Equation (1) involves two terms that minimize the disimilarity between the images and the atlas and the Sobolev norm of velocity fields *v*_*i*_; and Equation (2) shows the Lagrangian ordinary differential equations that define the model of deformation flow with the simulated time variable *s*. We use an iterative numerical approach through Algorithm 1 to solve this problem. The algorithm starts by assuming identity transformations *h*_*i*_, with an initial estimate of *I* obtained from weighted averaging of rigidly co-registered images *I*_*i*_. Symmetric deformable registration is performed between *I* and every *I*_*i*_

**Table:** 

Algorithm 1:
1. For each simulated time *s*, initialize *v*_*i*_ with Identity Transform;
2. For *i* = 1…*M* solve $${v}_{i}^{\ast }(x,t)=argmi{n}_{{v}_{i}(x,t)}[E(I(t),{I}_{i},{h}_{i})+{\Vert L{v}_{i}\Vert }^{2}]$$
3. Set optimal ${\bar{v}}^{\ast }(x,t)$ as
$${\bar{v}}^{\ast }(x,t)=\frac{1}{{{\rm{\Sigma }}}_{i={1}^{M}}{\bf{K}}(t-{t}_{i})}{{\rm{\Sigma }}}_{i=1}^{M}{\bf{K}}(t-{t}_{i}){v}_{i}^{\ast }(x,t)$$
where ${\bf{K}}\mathrm{(.)}$ is a kernel function.
4. Find the new $\bar{h}(x,t)$ based on $\bar{v}(x,t)$
5. Repeat until $\bar{h}(x,t)$ converges and ${\bar{I}}_{t}$ is obtained.

in each iteration, and the average deformation is computed by kernel-weighted averaging of the deformation fields.

**Figure 2:**
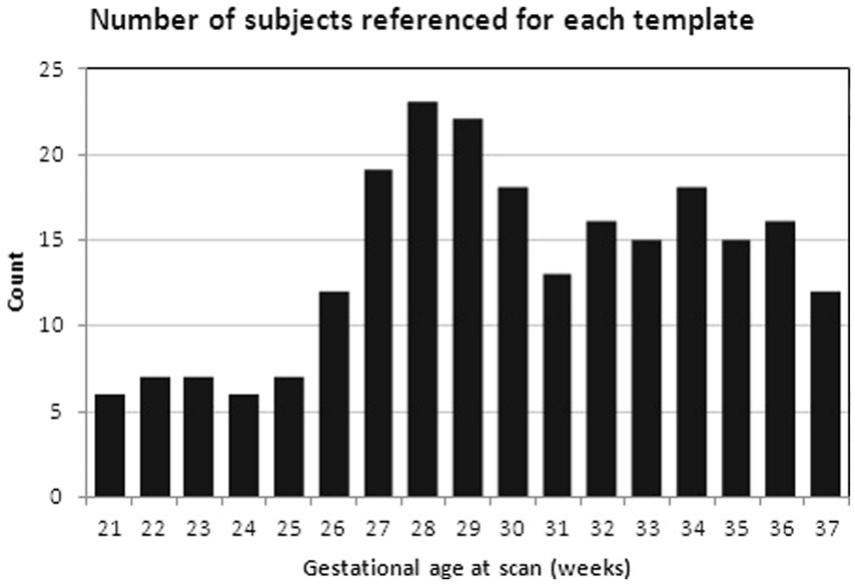
Frequency distribution of subjects contributed to atlas construction at each gestational age point in weeks. The number of subjects used in atlas construction at lower GAs was, on average, smaller than the numbers at higher GAs, which was acceptable as the fetal brain has less features and variability at lower GAs compared to higher GAs.

The algorithm converges in 5 to 10 iterations and generates a sharp anatomical image at a given age point *t*, that is an unbiased representative of the population anatomy at that age. The contribution of each subject to the atlas at any age point is proportional to the distance of the subject age to the atlas age point. We used a Gaussian kernel with standard deviation of 1. All kernel weights above 0.01 were retained and were normalized to fulfill the sum-of-unity property. Figure 2 shows the distribution of subjects contributed to each point of age for atlas construction. We used the negative of the cross correlation similarity metric between two images as the cost function in Equation (1). The minimization in step 2 of the algorithm was performed through symmetric diffeomorphic deformable registration using ANTS tools^60^ with greedy symmetric normalization, gradient step size of 0.05, Gaussian regularization (2, 0.05), and 100 × 100 × 20 maximum iterations.

### Atlas labeling and segmentation

As an average representation of normal anatomy, the spatiotemporal fetal brain MRI atlas delineates tissue characteristics of the fetal brain across gestation with relatively high signal-to-noise ratio, thus provides a relatively reliable framework for tissue-type structural segmentation and labeling. To generate atlas labels we started from the manually labeled ALBERTs neonatal brain atlases that have been generously distributed by their developers at brain-development.org^66, 67^. The ALBERTs templates include high-resolution T2-weighted MRI scans of 20 neonates manually labeled to 50 anatomically specified regions.

**Figure 3:**
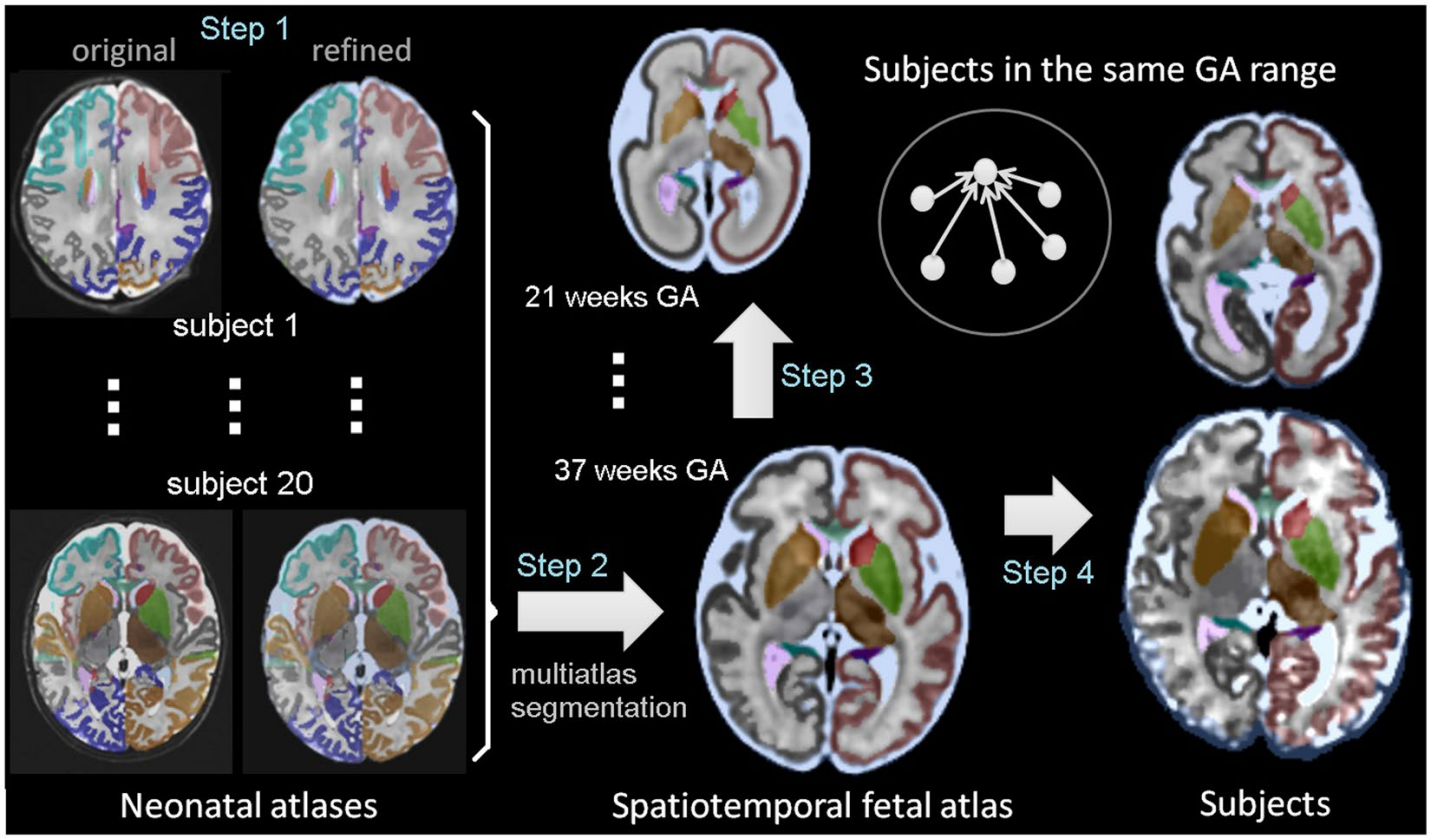
The procedure to generate atlas labels and segmentations: Step 1: labels and tissue-type segmentation of 20 neonatal ALBERTS atlases^66, 67^ were refined manually. Step 2: The segmented neonatal atlases were used to generate initial labels on the spatiotemporal fetal brain MRI atlas at higher GAs (35–37 weeks) through multiatlas segmentation using probabilistic label fusion^65^. Step 3: Fetal brain MRI labels were manually defined and propagated in iterations from the higher GAs to the lower GAs. Step 4: Atlases within one week of any query subject were used to generate an initial segmentation of that subject. The initial segmentations of all subjects within one week of the query subject were used to segment that subject.

We preprocessed the ALBERTs labels to prepare them for the purpose of segmenting our spatiotemporal fetal brain MRI atlas at late GAs. This included refinements to use an automatic atlas and intensity based segmentation algorithm to propagate labels from the ALBERTs atlases to the spatiotemporal fetal brain MRI atlas. This first step involved tissue-type segmentation to add labels for white matter, cortical gray matter, and extracerebral CSF and merging the cortical layer labels to improve registration and automatic segmentation. For initial tissue-type segmentation we constructed probability mass functions of 10 clusters through intensity clustering^74^. Each class label in the 10-class clustering was considered a tissue type which captured local and fine intensity information. The tissue types were then combined to guide CSF, gray matter, and white matter manual segmentations. As part of this process we also corrected some errors such as missed slice or region segmentations. The entire procedure used to generate atlas labels and individual subject segmentations is shown in Fig. 3 which includes examples of the neonatal atlases after tissue segmentation and manual refinement on the right.

In the second step, we used ANTS symmetric normalization with greedy optimization and the correlation ratio similarity metric^60^ to map the neonatal atlases to the late GA atlases of the fetal brain. We propagated the labels and used the probabilistic STAPLE method^65^ for label fusion. This is a multi-atlas segmentation method that incorporates local quality of probabilistic segmentations to propagate atlas labels from the refined ALBERTs atlases to the spatiotemporal fetal brain atlas at 35 to 38 weeks gestation only. In the third step, we manually generated labels on the fetal atlases and propagated them to lower GAs again using the probabilistic STAPLE method. Each round of manual segmentation of each atlas took between 1 to 5 days depending on the GA (i.e. the size and complexity of the brain). Labeled fetal MRI atlases at ages t + 2, t + 1 and t were used to generate labels for the atlas at age t−1. This procedure was repeated along with manual labeling to segment the entire age range of the spatiotemporal fetal brain MRI atlas at one week intervals.

Through manual segmentation the following structures (right and left, when applicable) were labeled on fetal brain MRI atlases: hippocampi, amygdala, fornix, cerebellum, brainstem, caudate nuclei, thalami, subthalamic nuclei, lentiform nuclei, corpus callosum, lateral ventricles, developing white matter, cortical plate, and cerebrospinal fluid (CSF). For most of these structures segmenters followed the segmentation protocol by Gousias *et al*.^66^, and consulted^75, 76^ as reference for labeling structures at lower gestational ages. Corpus callosum which appeared by hypointensity compared to nearby structures was segmented primarily on sagittal planes, and was repeatedly visualized in 3D and refined in other planes based on local contrast and its dark appearance. Ventrally it bounded with fornix which also appeared as hypointensity and was mainly segmented in the axial plane and refined in other planes. Hippocampi, which appeared darker than the surrounding developing white matter, were segmented primarily on sagittal planes and refined on coronal planes. Thalami segmentation started in the sagittal plane and was visualized and refined in the other planes. All other structures, including the lateral ventricles and cortical plate were segmented in multiple planes and visualized in 3D to ensure smooth and accurate segmentation. When applicable, we segmented three transient zones (left and right): subplate zone, intermediate zone, and ventricular zone, as described below.

Transient zones of the early developing brain^31, 34, 77, 78^ were distinguished on the spatiotemporal fetal brain atlas between 19 and 31 weeks gestation and gradually became less visible towards the end of the third trimester. While the subplate zone could be reliably segmented throughout this timeframe using published *in-vivo* imaging protocols^79, 80^, the ventricular and intermediate zones were variably distinguishable. The inner boundary of the subplate zone was defined by the transition from hyperintensity to hypointensity. This was done by adjusting the contrast and windowing around the medium intensity values. We initially manually segmented the subplate zone on the spatiotemporal atlas defined for 26 and 27 weeks gestation when it was well-visualized following the manual segmentation protocol described in ref. 79 and consulted additional reference MRI^19, 31, 81^ and pathology^75, 76^ images in cases where boundaries were difficult to define. For additional references on anatomy and a discussion on available resources we refer to the book chapter by Judas^82^. The segmentation was then propagated serially to younger and older GAs, manually editing each atlas before the next propagation. Above 27 weeks gestation, as the subplate gradually became more difficult to visualize, the segmentation relied mostly upon the atlas propagation. The ventricular zone was defined as the hypointense region adjacent to the ventricles and using^34^. The intermediate zone was defined by the outer boundary of the ventricular zone and the inner boundary of the subplate zone.

In the fourth step, we used the labeled spatiotemporal fetal brain MRI atlas along with a bootstrapping approach to segment the 81 subjects in our cohort. In this process, an initial segmentation was generated for each subject by using the three atlases closest to the GA of the subject. Then the initial segmentations of all subjects within one week of the GA of the query subject were used as new atlases to segment that subject (a bootstrapping strategy). This boosted the number of atlases and the accuracy of multi-atlas segmentation using probabilistic STAPLE^65^ which was particularly effective due to the use of estimated local quality of probabilistic segmentations of a relatively large number of atlases. After probabilistic atlas-based segmentation, we applied a Gaussian mixture model to correct for partial voluming effects between cortical gray matter and white matter, and cortical gray matter and CSF^48, 83^.

For quantitative evaluation of atlas-based segmentation, we used a leave-one-out strategy on the test set. Manual segmentation of the test subjects was performed in several rounds in different planes and took anywhere between 4 to 10 days depending on the age of the fetus. These subjects were also used as new and additional individual-subject atlases for the evaluation of multi-atlas segmentation. We used the probabilistic STAPLE segmentation approach and calculated the Dice Similarity Coefficient (DSC) for the test subjects. We compared the performance of segmentation using the spatiotemporal atlas and using the combination of the spatiotemporal atlas and individual-subject atlases. In the next section, we show the atlas, compare it to the fetal brain MRI atlas at brain-development.org, and present the results of automatic fetal brain MRI segmentation.

## Results

### The spatiotemporal fetal brain MRI atlas

**Figure 4:**
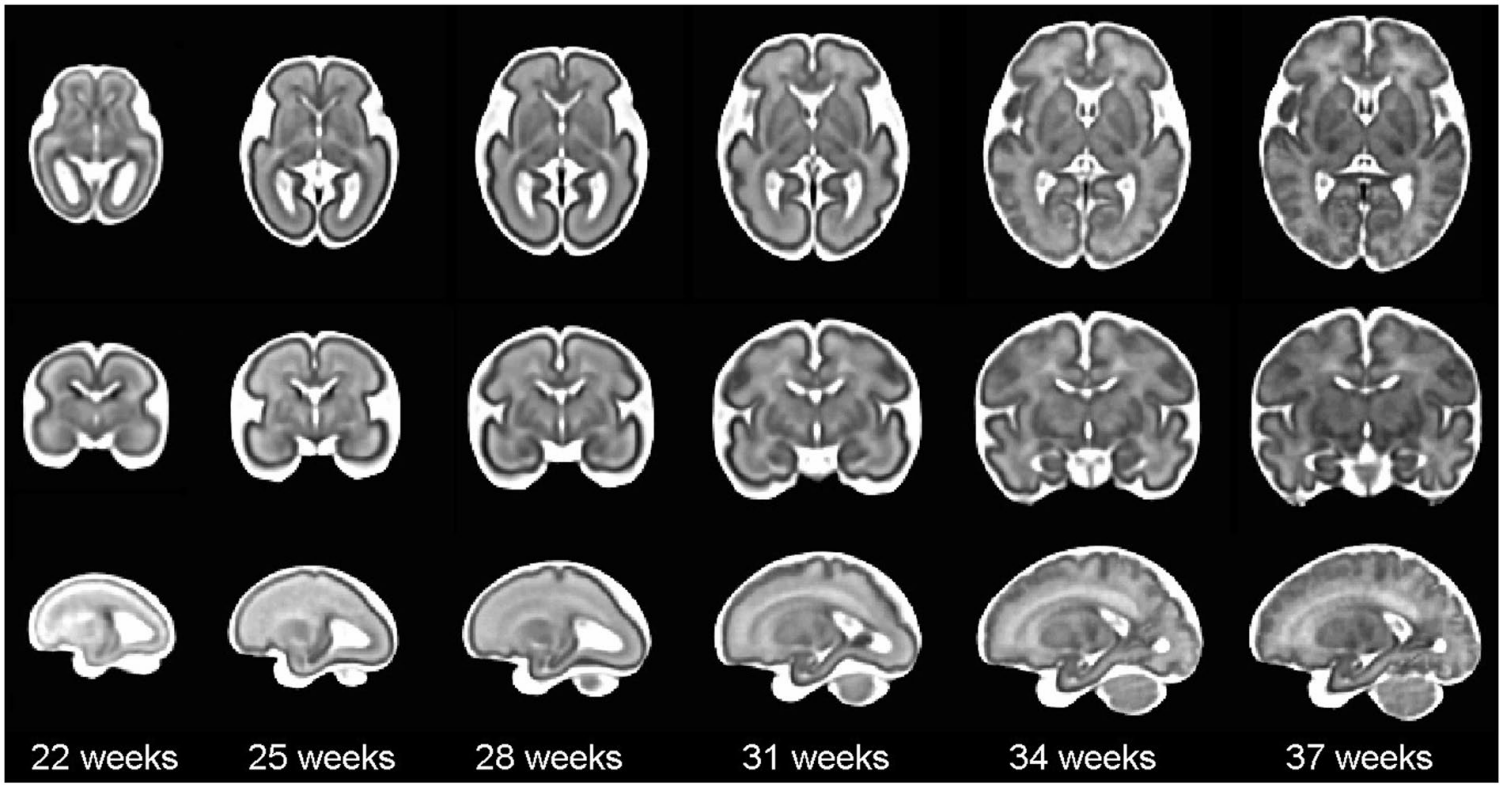
The spatiotemporal fetal brain MRI atlas (CRL fetal brain atlas) at six representative GAs: 22, 25, 28, 31, 34, and 37 weeks. Axial, coronal, and sagittal views of the atlas have been shown at each age point. Note that the spatiotemporal atlas construction process is a time-continuous process, therefore the atlas can be constructed at any continuous age point within the age range of the subjects used in the atlas construction process.

**Figure 5:**
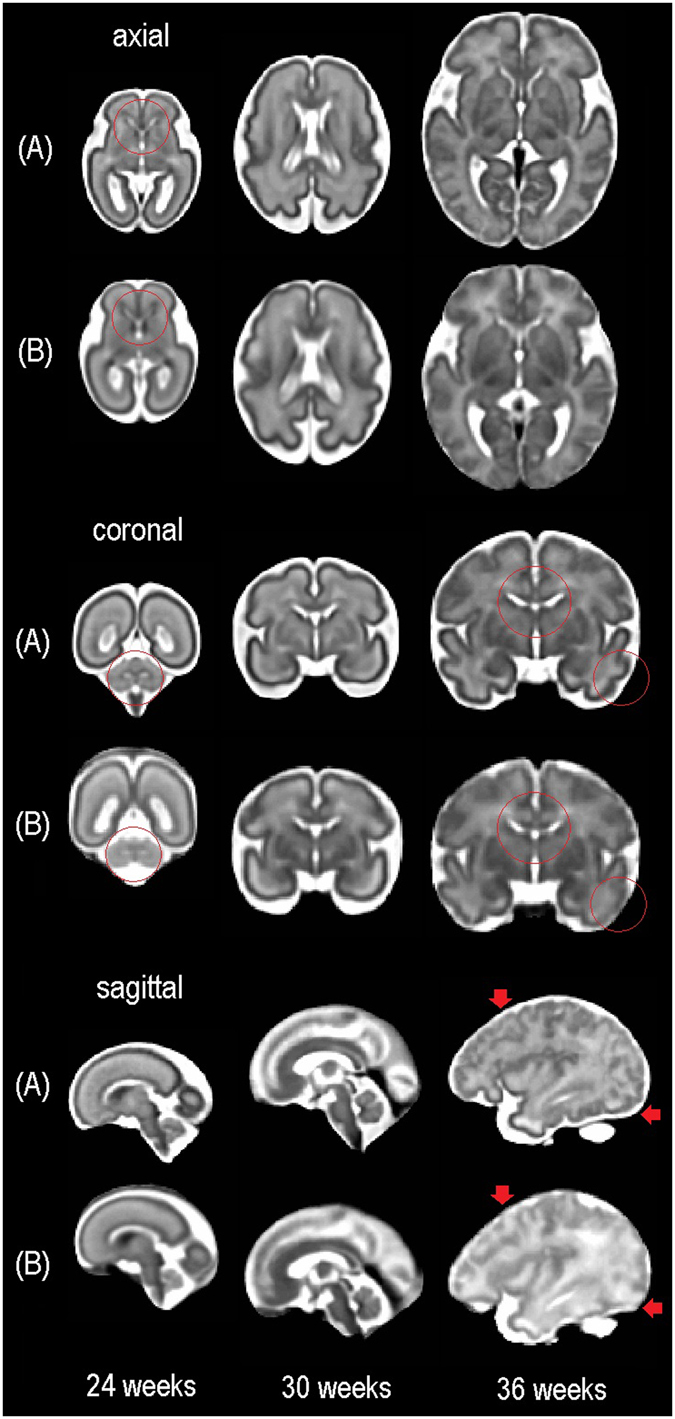
Visual comparison of our spatiotemporal fetal brain MRI atlas (**A**) and the atlas from brain-development.org (**B**). We did not register or resample the atlases to avoid artificial blur; we instead tried to compare the closest planes in each view at three ages (24 weeks, 30 weeks, and 36 weeks GA). The images in (**B**) are generally smoother than those in (**A**) but lack anatomic details compared to the images in (**A**). Red circles and markers point at some of the areas with relatively blurred anatomy on the atlas from brain-development.org but with more details on the CRL fetal brain atlas. Both atlases are available online.

The fetal brain MRI scans were pre-processed by the steps discussed in the Methods Section. Volumetric fetal brain MRI was reconstructed for all fetuses with an isotropic resolution of 1 *mm* ^3^ in 3D using robust super-resolution volume reconstruction^38^. The processed images were then used for spatiotemporal atlas construction through Algorithm 1. Figure 4 shows axial, coronal, and sagittal views of the spatiotemporal atlas across multiple GAs. Note that with Algorithm 1 an unbiased average atlas of the fetal brain anatomy is achieved at any given continuous age point, so these are only representative age points. The atlas (CRL fetal brain atlas), along with its labels, is available online at http://crl.med.harvard.edu/research/fetal_brain_atlas/ at one week intervals. Figure 5 compares the CRL atlas with the atlas accessed through brain-development.org in 2016. It is interesting to report that despite being based on different imaging at two different sites with different image reconstruction and processing methods, the two atlases comply very well. Side-by-side visual comparison in this figure suggests that the CRL atlas is sharper and better preserves anatomical details. Note that we did not register or resample the atlases to avoid inducing blur artifacts, and rather tried to compare the closest match between different planes.

### Atlas labels and segmentation

**Figure 6:**
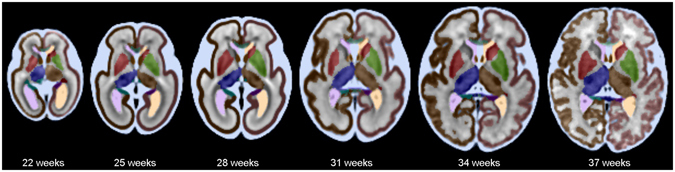
Tissue segmentation and structural labels defined and overlaid on the spatiotemporal fetal brain MRI atlas at six representative GAs: 22, 25, 28, 31, 34, and 37 weeks. The labels visible on these axial sections include the developing white matter, cerebrospinal fluid (CSF), corpus callosum, and left and right gray matter (cortical plate), ventricles, thalami, hippocampi, and lenticular and caudate nuclei.

We generated labels on the spatiotemporal fetal brain atlas by the process described in the Methods Section. Figure 6 shows tissue and anatomic structural labels on the spatiotemporal fetal brain MRI atlas. To evaluate automatic multi-atlas segmentation using the spatiotemporal atlas and the individual subject atlases, we used label propagation through symmetric diffeomorphic deformable registration^60^ between each subject’s anatomical image and atlases within one week of the subject’s GA. This was followed by label fusion in the subject’s anatomical space using the probabilistic label fusion approach^65^.

**Figure 7:**
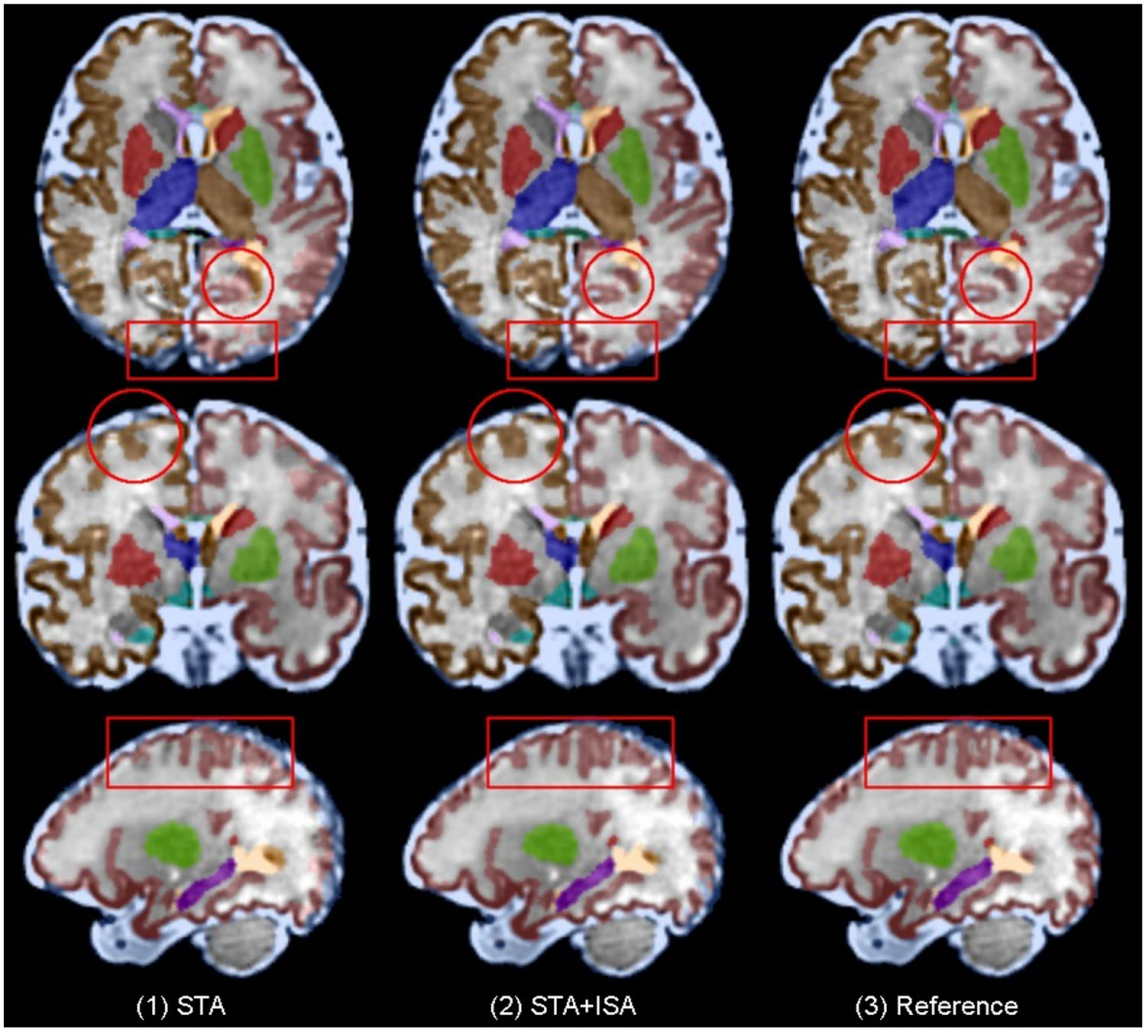
Visual assessment and comparison of atlas-based segmentation of fetal brain MRI using multiple atlases: (1) segmentation using the spatiotemporal fetal brain MRI atlas (STA), (2) segmentation using the combination of the spatiotemporal atlas and individual-subject atlases (STA + ISA), and (3) reference standard. The circles and squares point at some of the areas in which the methods performed differently. Overall the results were satisfactory. As expected due to the use of a larger number of atlases, slightly better performance was observed for STA + ISA. For quantitative comparison of methods (STA and STA + ISA) we relied on the analysis of average DSC metrics reported in Table 1. Labels that are visible on these images are CSF, corpus callosum, developing white matter, and left and right cortical plates, ventricles, thalami, hippocampi, amygdalae, and caudate nuclei.

We compared two scenarios for multi-atlas segmentation: 1) using spatiotemporal atlases (STA), and 2) using the combination of spatiotemporal atlases and the individual subject atlases (ISA + STA), all within one week of the query GA. Figure 7 shows visual comparison of the segmentations obtained from these methods compared to the reference on the right (3). Visual inspection of the results showed that multi-atlas segmentation performed fairly well in many areas, but there were also some errors highlighted by rectangles and circles. The results also indicated slight improvement by using individual-subject atlases in addition to the spatiotemporal atlases. This was expected and attributed in part to the use of a larger number of atlases and in part to the diversity in anatomy provided by ISAs compared to STAs. The number of STAs for each subject was 3 and the number of ISAs was between 0 and 2 depending on the age. Table 1 shows average DSC metrics for STA and STA + ISA for different structures. Overall, these values also indicate slight improvement in performance by using ISAs in addition to STAs. The improvement in performance was relatively large for corpus callosum.

**Table 1:** Comparing average DSC metrics for multi-atlas segmentation using the spatiotemporal atlases (STA) and the combination of STA and individual subject atlases (STA + ISA).

	ThalL	ThalR	CC	VentL	VentR	Brainstem	CPL	CPR	WML	WMR	CSF
STA	0.916	0.931	0.677	0.930	0.938	0.972	0.925	0.922	0.872	0.877	0.900
STA + ISA	0.918	0.933	0.766	0.932	0.933	0.974	0.927	0.924	0.876	0.880	0.901

ThalL: left thalamus, ThalR: right thalamus, CC: corpus callosum, VentL: left lateral ventricle, VentR: right lateral ventricle, Brainstem: brainstem, CPL: left cortical plate, CPR: right cortical plate, WML: left white matter, WMR: right white matter, and CSF: cerebrospinal fluid. In average higher DSC values were obtained from STA + ISA, which was expected due to the use of larger number of atlases. The difference was particularly high for CC.

**Figure 8:**
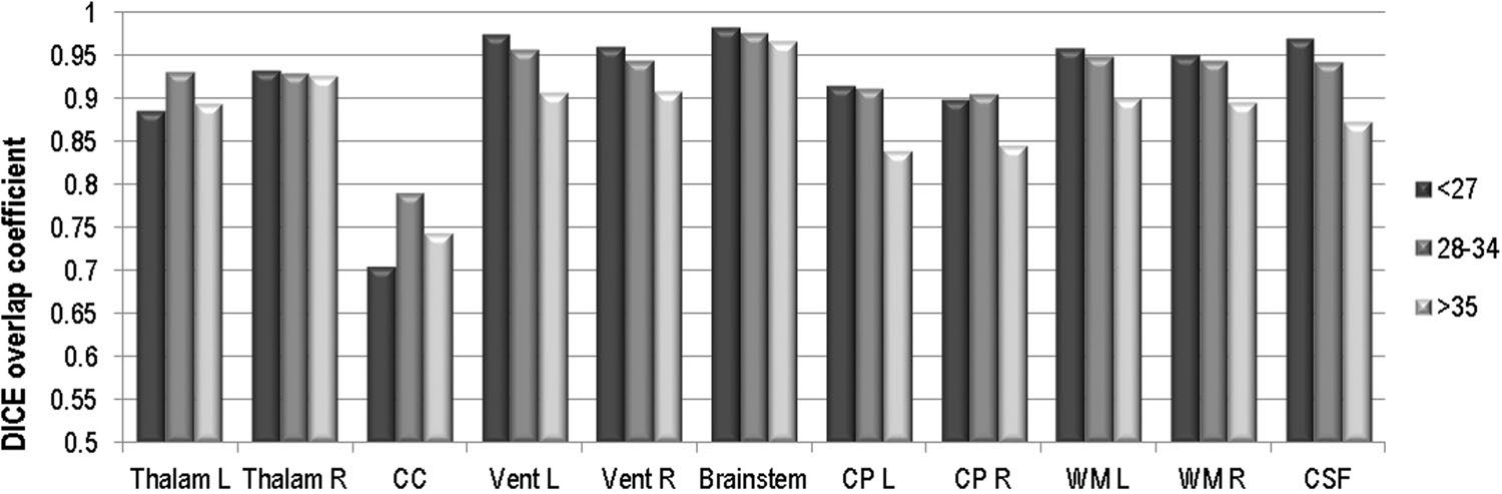
DSC metrics computed for brain tissue and structures for leave-one-out automatic multi-atlas segmentation evaluation applied to seven subjects with reference manual segmentations (the test set), averaged in three GA ranges: less than 27 weeks, between 28 and 34 weeks, and more than 35 weeks. Overall, the results indicated that relatively accurate automatic segmentation of the main brain structures and tissue were achieved by using the developed spatiotemporal fetal brain MRI atlas, symmetric diffeomorphic deformable registration for label propagation^60^, and probabilistic STAPLE^65^ for label fusion. Labels are: Thalam L: left thalamus, Thalam R: right thalamus, CC: corpus callosum, Vent L: left lateral ventricle, Vent R: right lateral ventricle, Brainstem, CP L: left cortical plate, CP R: right cortical plate, WM L: left developing white matter, WM R: right developing white matter, and CSF: cerebrospinal fluid.

Figure 8 shows quantitative automatic multi-atlas segmentation results based on the DSC metric averaged for the test subjects in three different GA groups. This analysis indicated that by using multiple atlases at 1 week intervals around the query subject GA, accurate automatic segmentation of fetal brain MRI was achieved with DSC values around 0.84 to 0.91 for the cortical plate, between 0.89 and 0.95 for the developing white matter, around 0.9 to 0.95 for the ventricles, CSF, and brainstem, and between 0.7 and 0.8 for the corpus callosum, which was very challenging due to its narrow shape and the effect of partial voluming. It was also observed that in average more accurate segmentations were obtained for fetuses at lower GAs and the task became more challenging as the brain evolved to have more complex shape and structures. This trend was specifically observed in the DSC of the larger tissue types like cortical plate, developing white matter, and CSF. On the other hand, automatic segmentation of small and narrow structures such as corpus callosum appeared particularly challenging at lower GAs (DSC of 0.7 at <27 weeks GA). We attribute this to the thin shape and size of these structures that is not much larger than the effective spatial resolution of fetal MRI. Consequently, partial voluming affects the appearance and contrast of these structures and reduces the accuracy of deformable registration and in turn the accuracy of label propagation and segmentation.

## Discussion and Conclusion

In this study, we generated a 4D spatiotemporal MRI atlas of the fetal brain from reconstructed 3D brain MRI of 81 healthy fetuses scanned at different GAs. The prerequisite for the construction of this 4D atlas was reconstruction of individual 3D fetal brain MRIs from multiplanar stacks of 2D slice acquisitions, which was performed using a robust super-resolution volume reconstruction algorithm^38^. In contrast to previous works that focused on probabilistic atlas construction which relied upon manual segmentations of original data^44, 48, 49, 51–53, 57^, we focused on the construction of a deformable spatiotemporal atlas by integrating kernel regression in time (age) with symmetric diffeomorphic deformable registration in space. This method allowed effective compensation of spatiotemporal variability between subjects scanned at different GAs and led to sharp atlases with anatomical details that helped us generate tissue and anatomical labels on the atlas. Atlas construction using kernel regression based on symmetric diffeomorphic deformations guarantees inverse consistent large deformations that reduce asymmetric bias. These properties cannot be easily achieved by free-form deformations. On top of the technical differences mentioned above, the CRL atlas covers a wider GA range than the atlases developed by Habas *et al*.^44^, Dittrich *et al*.^53^, and Zhan *et al*.^55^. The CRL atlas is most similar in nature to the atlas that is available at brain-development.org^52^ (compared in Fig. 5).

Our atlas labeling procedure, illustrated in Fig. 3 and discussed in Section Atlas Labeling and Segmentation, relied upon two main components: probabilistic STAPLE label fusion^65^ which was used in steps 2 to 4 (Fig. 3) for iterative label propagation, and manual segmentation which was the most time consuming and laborious part of the procedure. We took a major step forward by labeling subcortical brain structures and the developing zones of the brain when they were reliably visible and distinguishable on the atlas. It is important to note the intrinsic limited spatial resolution and contrast of *in-vivo* fetal MRI and its effect on the resolution and accuracy of this atlas and its labels. The CRL atlas is aimed to facilitate and improve *in-vivo* fetal brain MRI analysis. It is not comparable to and should not replace high-resolution atlases based on histology or *ex-vivo* MRI. For detailed high-resolution pictures and description of the anatomy we refer to the books and papers based on histology and *ex-vivo* imaging^31, 34, 75–78, 82^. There are certainly many small structures or substructures that could not be reliably visualized and labeled on this atlas. The smaller structures also should be used with caution. Many of these structures were visualized in 3D on the spatiotemporal fetal brain MRI atlas because of the inherent boost in signal due to temporal averaging of the anatomy of samples from the population. The 3D appearance of these structures on individual *in-vivo* fetal brain MRI scans depends on the quality of the scans (which is mainly affected by fetal and maternal motion) and the reconstruction procedure. The use of a robust motion correction and reconstruction procedure is crucial.

The deformable spatiotemporal fetal brain MRI atlas, presented here, characterizes normal fetal brain development. The atlas may be used as a reference for registration and spatial normalization in groupwise and longitudinal studies. The atlas with labels may be used for atlas-based segmentation in volumetric or morphometric analysis, or as a reference for connectivity analysis. As a direct application of the developed atlas, we used it for automatic multi-atlas segmentation of reconstructed fetal brain MRI. Qualitative and quantitative results, based on visual inspection and DSC, calculated and reported on the test set, indicate that accurate automatic fetal brain MRI segmentation can be achieved by using a robust multi-atlas segmentation approach. As expected^65, 84, 85^, we observed that higher number of atlases and more diverse pool of atlases resulted in more accurate multi-atlas segmentations. It was also observed that the segmentation accuracy could be relatively low (DSC around 0.7–0.8) for small and narrow structures such as corpus callosum, especially at lower GAs when the size of these structures were comparable to the effective spatial resolution of fetal MRI. As mentioned earlier, small and narrow structures can be obscured and affected by partial voluming on reconstructed *in-vivo* fetal MRI scans, therefore may not be reliably segmented even with a robust multi-atlas segmentation strategy. This is not a limitation of the atlas but is mainly a limitation of *in-vivo* fetal MRI. The accuracy of the segmentation of these structures depends on the quality of the original scans and the reconstruction and post-processing procedures, as the lack of contrast will adversely affect the performance of deformable registration and label propagation. As a result, atlas-based segmentation of small and narrow structures, such as hippocampus, corpus callosum, and amygdala, should be performed cautiously. Fetal MRI analysis has advanced significantly in the recent years^68^. With widespread use of advanced imaging, reconstruction tools, and resources like the developed atlases, we expect major improvements in efficiency and accuracy of large-scale studies on early human brain development.
